# Genetic Influence of *CCDC63* Polymorphisms on Alcohol-Induced Dyslipidemia in a Korean Cohort

**DOI:** 10.3390/ijms27052134

**Published:** 2026-02-25

**Authors:** Yu-Na Kim, Sung Won Lee, Hyun-Seok Jin, Sangwook Park

**Affiliations:** 1 Department of Biomedical Laboratory Science, College of Health and Biomedical Services, Sangji University, Wonju 26339, Republic of Korea; uim2878@sj.sangji.ac.kr (Y.-N.K.); sungwonlee@sangji.ac.kr (S.W.L.); 2 Department of Biomedical Laboratory Science, College of Life and Health Sciences, Hoseo University, Asan 31499, Republic of Korea; jinhs@hoseo.edu

**Keywords:** alcohol interaction, *CCDC63*, dyslipidemia, genetic stratification, pleiotropy

## Abstract

While chronic alcohol consumption is an established risk factor for lipid metabolic dysregulation, the underlying genetic mediators remain largely elusive. This study investigated the synergistic impact of *CCDC63* (coiled-coil domain containing 63) polymorphisms and alcohol intake on dyslipidemia risk within a Korean cohort. Leveraging data from the KARE study (*N* = 6655; 4327 dyslipidemia cases vs. 2328 controls), we analyzed SNPs across the *CCDC63* locus via Affymetrix SNP Array 5.0. Logistic regression, adjusted for age and sex, was performed to evaluate genotype–phenotype association and gene–environment interactions induced by alcohol exposure duration. Three intronic variants (rs10849915, rs11065756, and rs2238149) were significantly associated with dyslipidemia (OR ≥ 1.15, *p* < 0.005). Notably, stratified analysis revealed a clear gene–environment interaction. In ever-drinkers, the G-allele of rs10849915 was significantly associated with a higher risk of dyslipidemia (OR = 1.23, *p* < 0.05), significantly lower γ-GTP levels (β = −8.08), and reduced HDL (β = −1.42). However, no such genetic associations were observed in the non-drinking group (*p* > 0.05 for all traits). Our findings demonstrate that *CCDC63* variants specifically modulate lipid metabolism and hepatic enzyme levels in an alcohol-dependent manner. The paradoxical association—lower γ-GTP yet higher dyslipidemia risk in drinkers—suggests that *CCDC63* plays a critical role in the complex interplay between alcohol exposure and systemic lipid homeostasis.

## 1. Introduction

Dyslipidemia, or abnormal blood lipid levels, represents a major modifiable risk factor for cardiovascular disease, affecting approximately 40% of Korean adults [1,2]. Alcohol consumption considerably influences lipid metabolism through complex interactions with genetic background and other environmental factors [3,4]. Moderate alcohol intake may increase HDL cholesterol levels, while heavy or chronic consumption promotes hypertriglyceridemia and fatty liver disease [5,6]. Research in Asian populations exhibits distinct patterns of alcohol-related dyslipidemia, with genetic factors explaining much of the individual variation in metabolic responses [7,8]. Genome-wide association studies have found various genetic regions linked to alcohol consumption and lipid metabolism traits across diverse ethnic populations [9,10]. The *CCDC63* gene has consistently shown links to alcohol consumption in African American, European, and East Asian groups. In particular, the rs10849915 variant is strongly associated with both weekly alcohol intake and the frequency of drinking [10,11,12,13,14,15,16]. However, it remains unknown whether the *CCDC63* gene alters lipid metabolic responses under chronic alcohol exposure.

eQTL (expression quantitative trait locus) resources imply that *CCDC63* variants modulate transcription in tissues that are key to lipid homeostasis, such as the liver and skeletal muscle [17,18]. However, direct mechanistic studies are still warranted. Compared to studies focused on people of European descent, research on East Asians, including Koreans, provides additional insights thanks to distinct patterns in linkage disequilibrium and allele frequencies [14,19]. Furthermore, susceptibility mechanisms distinct from those elicited by steady, moderate consumption in Western populations may be triggered by episodic heavy drinking, which is common in Korea [20,21]. A comprehensive understanding of how *CCDC63* polymorphisms influence the association of the alcohol–dyslipidemia relationship could inform precise prevention strategies in communities with high rates of alcohol [22]. Accordingly, we analyzed a large Korean cohort from the Korean Association REsource (KARE) to evaluate associations between *CCDC63* variation and dyslipidemia, with a specific focus on gene–environment interactions by alcohol-exposure duration. We speculated that differences in the *CCDC63* gene might impact both lipid metabolism and liver function associated with alcohol intake. We suggested that chronic alcohol exposure may influence the risk of dyslipidemia differently. These SNPs likely share linkage disequilibrium with each person’s genotype. We also explored whether these genetic effects might be explained by intermediate phenotypes such as liver enzyme levels or markers of inflammation.

## 2. Results

### 2.1. Participant Characteristics and Lipid Profiles

Our analysis included 6655 participants—47.7% male, with mean age 52 years and BMI 24.6 kg/m^2^ (Table 1). Comparing dyslipidemia cases to healthy controls revealed both demographic and metabolic differences. Cases were more likely to be male (52.4% vs. 39.0%, *p* < 0.001), slightly older (52.7 vs. 51.1 years, *p* = 0.013), and had higher BMI (25.2 vs. 23.3 kg/m^2^, *p* = 0.003). Lipid measurements diverged more sharply. Cases averaged 196 mg/dL for total cholesterol vs. 169 mg/dL in controls. Triglycerides showed an even larger gap—185 mg/dL in cases compared to 99 mg/dL in controls, nearly a twofold difference. LDL cholesterol was elevated in cases (120 vs. 98 mg/dL), while HDL cholesterol showed the reverse pattern: cases had lower levels at 39 mg/dL vs. 51 mg/dL in controls. All lipid parameters differed significantly between groups (*p* < 0.001), confirming that our case definition successfully separated individuals with dyslipidemia from those with healthy lipid profiles.

Alcohol consumption and smoking characteristics are detailed in Supplementary Table S3. Current drinkers comprised 46.1% of participants, with cases showing higher total alcohol intake (20.9 vs. 18.7 g/day, *p* = 0.039). Soju and spirits consumption were significantly associated with dyslipidemia (*p* = 0.006 and *p* < 0.001, respectively). Current smokers represented 26.0% of the cohort, with significantly higher prevalence in the dyslipidemia group (71.0% vs. 29.0%, *p* < 0.001).

### 2.2. Genomic Landscape of CCDC63 and Dyslipidemia Risk

To determine if *CCDC63* plays a role in lipid metabolism, we examined six intronic variants within the gene. Our analysis identified a clear association between dyslipidemia and three specific markers—rs11065756, rs10849915, and rs2238149—all of which met strict statistical criteria (*p* < 0.005; Table 2). What stood out was the striking consistency in their effect sizes; each variant carried a nearly identical risk, with odds ratios tightly clustered between 1.15 and 1.16. The three variants showed consistent effects. Each had nearly the same risk increase, with odds ratios between 1.15 and 1.16. Linkage disequilibrium analysis confirmed that these three SNPs form a haplotype block. Using the KARE cohort, we found that rs10849915 and rs11065756 are in near-perfect LD (r^2^ > 0.97), while rs2238149 shows moderate LD with the other variants (r^2^ = 0.75). This LD structure explains their similar allele frequencies (15.5–18.5%) and consistent effect sizes (OR 1.15–1.16), suggesting these variants tag a single underlying causal signal. Genotype-specific analysis for rs10849915 showed dyslipidemia prevalence of 63.8% in AA homozygotes (*N* = 4484), 67.4% in AG heterozygotes (*N* = 1914), and 68.8% in GG homozygotes (*N* = 221), demonstrating a dose-dependent increase consistent with the additive model (Supplementary Table S4).

### 2.3. Replication of rs10849915 Associations

The rs10849915 variant was previously identified in genome-wide studies of alcohol consumption. We confirmed this association in our Korean cohort [13]: the G allele showed a strong inverse relationship with alcohol intake (β = −0.31 ± 0.02, *p* = 1.31 × 10^−60^, Table 3). The consistency across populations—including European, African American, and now Korean samples—supports a genuine biological signal rather than population-specific artifacts.

### 2.4. Associations with Liver Enzymes and Lipid Markers

We examined whether the three *CCDC63* variants associate with liver enzymes and lipid levels (Table 4). Linear regression revealed significant associations with multiple biomarkers.

The strongest effects appeared for γ-GTP. All three variants correlated with lower γ-GTP levels, with effect sizes ranging from −6.99 to −7.55 U/L (*p* values from 2.07 × 10^−8^ to 8.67 × 10^−8^). ALT and AST showed similar negative associations, though with smaller magnitudes. HDL cholesterol also decreased with the minor alleles.

For rs10849915, the associations included ALT (β = −1.18 ± 0.51, *p* = 0.022), AST (β = −1.20 ± 0.37, *p* = 0.001), γ-GTP (β = −6.99 ± 1.29, *p* = 5.68 × 10^−8^), and HDL cholesterol (β = −0.87 ± 0.29, *p* = 0.002). The particularly strong γ-GTP association is notable because this enzyme serves as a biomarker for alcohol consumption, linking back to *CCDC63*’s established role in drinking behavior. Combined with the HDL findings, these results suggest *CCDC63* influences both alcohol processing and lipid metabolism.

### 2.5. Distinct Genetic Effects of Alcohol Consumption

To determine whether these genetic associations depend on alcohol exposure itself, we stratified participants by ever-drinking status (Supplementary Table S1). In ever-drinkers (current and former drinkers combined, *N* = 3485) the G-allele of rs10849915 maintained its significant association with dyslipidemia risk (OR = 1.23, *p* = 0.0074), lower γ-GTP levels (β = −8.08, *p* = 0.0013), and reduced HDL-cholesterol (β = −1.42, *p* = 6.58 × 10^−5^). Among non-drinkers, these relationships disappeared entirely—no significant associations were detected for any trait (*p* > 0.05). This divergence confirms that the metabolic impact of *CCDC63* variants is selectively triggered by alcohol exposure rather than being an intrinsic lipid defect.

### 2.6. Effects of Drinking Duration on Dyslipidemia Risk by Genotype

**Figure 1:**
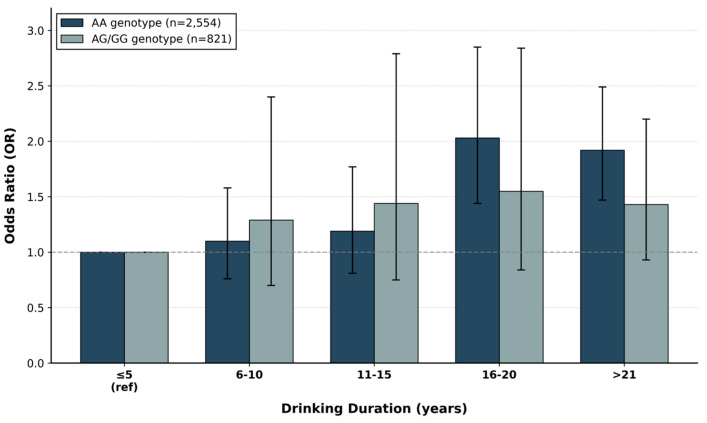
Dyslipidemia risk and drinking duration stratified by rs10849915 genotype. Odds ratios (OR) for dyslipidemia across drinking duration categories, stratified by rs10849915 genotype. Within each genotype group, the ≤5 years category serves as the reference (OR = 1.00). Dark bars represent AA homozygotes (*N* = 2554); light bars represent AG/GG heterozygotes (*N* = 821). Drinking duration categories: <5 years (reference), 6–10 years, 11–15 years, 16–20 years, and >21 years. Error bars indicate 95% confidence intervals. Dashed horizontal lines indicate OR = 1.0 (no effect). Estimates from logistic regression models adjusted for age, sex, body mass index, and gamma-glutamyl transferase levels.

We examined whether drinking duration affects dyslipidemia risk differently depending on rs10849915 genotype (Figure 1, Table 5). AA homozygotes (*N* = 2554) showed a striking pattern. Dyslipidemia risk increased progressively with drinking duration, peaking at 16–20 years (OR = 2.03, 95% CI: 1.44–2.85, *p* < 0.001 versus ≤5 years). Risk remained elevated beyond 21 years (OR = 1.92, 95% CI: 1.47–2.49, *p* < 0.001). In contrast, individuals carrying at least one G allele (AG/GG, *N* = 821) showed more modest increases—from OR 1.29 at 6–10 years (*p* = 0.04) to OR 1.55 at 16–20 years (*p* = 0.02). The effect magnitudes were roughly half those seen in AA individuals. A crossover pattern emerged from the interaction analysis. With short-term drinking (≤15 years), AG/GG genotypes associated with higher risk. However, prolonged exposure (>15 years) shifted the burden toward AA individuals, who became substantially more vulnerable. BMI showed consistent effects across both genotypes (AA: OR 1.33, AG/GG: OR 1.29, both *p* < 0.001). Age was significant only among AG/GG genotypes (OR 1.13, *p* < 0.001).

### 2.7. Sex-Specific Genetic Effects

Sex-stratified analysis revealed substantial differences in rs10849915 associations between males and females (Table 6). For dyslipidemia, the genetic effect appeared exclusively in males. Men carrying the minor allele faced increased risk (OR = 1.24, *p* = 0.003), while women showed no significant association (OR = 1.07, *p* = 0.273). Both sexes exhibited significant relationships with alcohol consumption, though males demonstrated stronger effects (β = −0.40, *p* = 2.71 × 10^−74^ versus β = −0.23, *p* = 1.66 × 10^−22^). Hepatic enzyme associations followed the same pattern. Males showed significant negative correlations across all three markers: ALT (β = −1.45, *p* = 0.012), AST (β = −1.98, *p* = 0.002), and γ-GTP (β = −13.02, *p* = 2.16 × 10^−7^). The γ-GTP effect proved particularly robust. Females demonstrated weaker associations—ALT (β = −0.97, *p* = 0.031) and γ-GTP (β = −1.60, *p* = 0.015) reached marginal significance, while AST did not (β = −0.51, *p* = 0.181). Lipid parameters diverged sharply between sexes. HDL cholesterol decreased substantially in males (β = −1.86, *p* < 0.001) but showed no effect in females (β = 0.06, *p* = 0.902). Triglycerides exhibited a similar male-specific pattern (male: β = −9.83, *p* = 0.006; female: β = 2.19, *p* = 0.391). Across all metabolic traits examined, rs10849915 associations appeared consistently stronger in males.

### 2.8. CCDC63 Tissue Expression Profile

**Figure 2:**
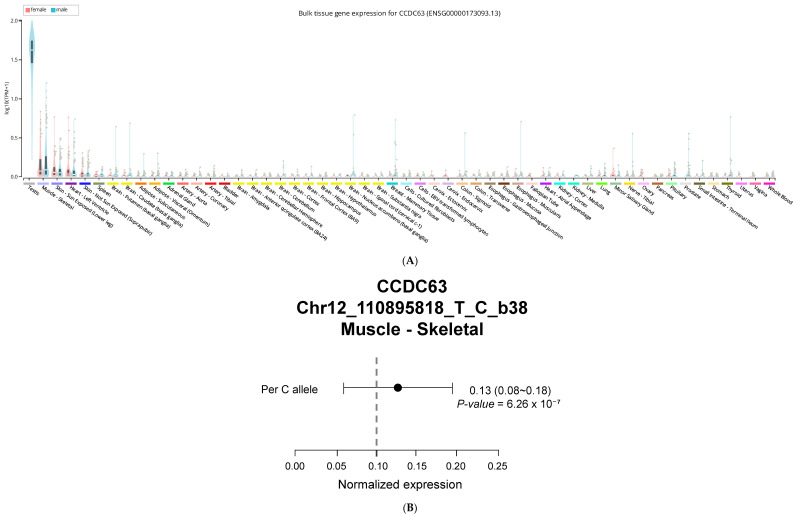
Tissue-specific expression profile of *CCDC63*. (**A**) Gene expression levels of *CCDC63* (ENSG00000173093.13) across 54 human tissues from the GTEx Portal (v10). Expression values shown as log-transformed transcripts per million [log(TPM + 1)] to stabilize variance across tissues. Highest expression observed in testis and skeletal muscle. Data accessed 7 November 2025. TPM, transcripts per million. Data for these analyses were retrieved from: [https://gtexportal.org/home/gene/ENSG00000173093.13 (accessed on 7 November 2025)] the GTEx Portal on 11/07/2025. (**B**) Forest plot showing the eQTL effect of rs10849915 (Chr12:110895818, T/C, GRCh38) on *CCDC63* expression in skeletal muscle. The T allele serves as the reference. The C allele shows a significant positive effect on expression (normalized effect size [NES] = 0.13, 95% CI: 0.08–0.18, *p* = 6.26 × 10^−7^). The filled circle represents the point estimate; horizontal lines indicate the 95% confidence interval. The C allele in GTEx (GRCh38) corresponds to the G allele in our GRCh37-based Korean dataset, which is associated with increased dyslipidemia risk (OR = 1.15). Data from GTEx Portal v10 (accessed 7 November 2025; dbGaP Accession phs000424.v10.p2).

CCDC63 showed highest expression in testicular tissue, followed by skeletal muscle according to GTEx v10 (Figure 2). Among other tissues, moderate expression appeared in adipose tissue and liver. The rs10849915 variant functions as an expression quantitative trait locus (eQTL) for CCDC63 exclusively in skeletal muscle tissue (*p* = 6.26 × 10^−7^, *N* = 816). The effect allele showed a positive normalized effect size (NES = 0.13), indicating increased CCDC63 expression in a dosage-dependent manner. No significant eQTL effects were detected in testis, adipose, or liver tissues despite measurable CCDC63 expression in these organs. This muscle-specific regulatory pattern suggests that rs10849915 resides within or near a tissue-selective enhancer element. GTEx Portal reports this variant using GRCh38 coordinates (chr12:110,895,818) with T/C allele designations, where the C allele increases CCDC63 expression (NES = +0.13). In our GRCh37-based Korean dataset (chr12:111,333,622), we use A/G notation where the G allele corresponds to the GTEx C allele.

## 3. Discussion

We confirmed the *CCDC63*-alcohol connection that previous genome-wide studies identified. In our Korean cohort, the G allele at rs10849915 had a strong negative link to alcohol consumption (β = −0.31 ± 0.02, *p* = 1.31 × 10^−60^). This matches findings from other populations [10,23]. The same three variants—rs10849915, rs11065756, and rs2238149—affected multiple traits. They associated with dyslipidemia, as well as alcohol-related markers: liver enzymes (ALT, AST, andγ-GTP) and HDL-cholesterol. This pattern suggests *CCDC63* connects how the liver processes alcohol with lipid metabolism. All three variants linked to lower HDL cholesterol. The effect was strongest for rs11065756 (β = −0.91 ± 0.29, *p* = 1.48 × 10^−3^). The clinical impact is clear. Each copy of the risk allele lowers HDL by 0.91 mg/dL. This effect, though modest, may contribute to cardiovascular risk given the established inverse relationship between HDL levels and cardiovascular disease [24]. Genetic background determined who developed dyslipidemia with prolonged drinking. AA homozygotes faced escalating risk—about 23% increase per decade of consumption. In contrast, people carrying at least one G allele (AG/GG genotypes) stayed relatively protected regardless of drinking duration. This gene-by-alcohol interaction opens doors for personalized risk assessment based on rs10849915 genotype [25]. To understand how rs10849915 might exert these effects, we examined the GTEx expression database and found that CCDC63 is directly regulated, especially in skeletal muscle. We also observed notable activity in the liver and fat tissue. Gene regulation that occurs in specific tissues serves as a bridge connecting genetic differences to how metabolism functions [17,26].

The GTEx database shows that rs10849915 affects CCDC63 expression in muscle (NES = 0.13, *p* = 6.26 × 10^−7^). This variant appears as C/T in GTEx (GRCh38) and A/G in our Korean dataset (GRCh37). The C allele in GTEx matches our G allele—both increase muscle CCDC63 expression. Higher expression makes sense given the G allele’s link to dyslipidemia (OR = 1.15). More CCDC63 in muscle could interfere with lipid handling.

Our finding that *CCDC63* variants associate with lower liver enzymes yet higher dyslipidemia risk in drinkers represents a notable metabolic paradox. This pattern closely resembles the well-known effects of the *ALDH2* rs671 polymorphism in East Asians, where individuals with reduced alcohol processing capacity often exhibit lower hepatic stress markers (γ-GTP) but miss the beneficial HDL-raising effect typically induced by ethanol consumption [27,28]. In our cohort, the lack of association in non-drinkers strongly supports the hypothesis that the G-allele’s impact on lipid profiles is a downstream consequence of altered alcohol-mediated metabolism. While the G-allele appears ‘protective’ of the liver (lower γ-GTP), the resulting decrease in HDL cholesterol levels leads to a higher clinical probability of dyslipidemia (OR = 1.23). This underscores that ‘genetically healthier’ liver enzyme levels do not always translate to a lower cardiovascular risk profile in the context of alcohol consumption (Table 4). The skeletal muscle-specific eQTL pattern provides mechanistic insight into the male-predominant associations observed for rs10849915 (Table 6). Men typically maintain 30–40% greater muscle mass than women, with regional differences being more pronounced in the upper body (40% difference) compared to the lower body (33% difference) [29]. This sex difference in muscle mass distribution potentially amplifies any muscle-based metabolic effects of *CCDC63* variants. The absence of significant eQTL activity in adipose or liver tissues—tissues where sex differences in mass and function are less pronounced—further supports skeletal muscle as the primary tissue mediating this variant’s metabolic effects. Testosterone-regulated transcription in muscle tissue may enhance the variant’s impact on systemic metabolism in males. GTEx reports this variant using T/C allele designations on GRCh38, while our Korean dataset uses A/G notation on GRCh37. The GTEx C allele (effect allele, NES = +0.13) corresponds to our G allele (risk allele, OR = 1.15). The tissue-specific expression of *CCDC63* helps clarify how one genetic variant can influence metabolism throughout the entire body. The strong effects observed in males are reasonable, considering how actively this gene functions in the testes. The transcription process influenced by testosterone likely enhances the metabolic impact of this variant [30]. The male-predominant associations warrant further mechanistic consideration. Intrinsic sex differences in alcohol pharmacokinetics include lower gastric alcohol dehydrogenase (ADH) activity in women, resulting in higher systemic alcohol exposure per unit consumed [31]. Paradoxically, this would predict stronger genetic effects in women if systemic alcohol concentration were the primary driver. The observed male-predominant pattern therefore suggests that *CCDC63*–alcohol interactions operate through tissue-specific mechanisms—particularly the skeletal muscle eQTL—rather than systemic alcohol levels. Several additional factors likely contribute to the stronger effects observed in males. First, men in our Korean cohort consumed substantially more alcohol than women, providing greater opportunity for gene–environment interaction to manifest phenotypically. Second, sex hormones may directly modulate *CCDC63* function or its downstream metabolic effects. Testosterone, which regulates muscle protein synthesis and lipid oxidation, may amplify the metabolic consequences of *CCDC63* variation in skeletal muscle [30]. Conversely, estrogen influences hepatic lipid metabolism through estrogen receptor-α signaling and may buffer against alcohol-induced metabolic perturbations in premenopausal women [32]. These hormonal influences could explain why *CCDC63* effects on dyslipidemia and HDL-cholesterol were significant only in males (Table 6). The clinical implications of these sex-specific findings deserve attention. Genetic risk assessment incorporating *CCDC63* variants may provide greater predictive value in male patients, while utility in females requires further investigation with detailed hormonal profiling and stratification by menopausal status.

Our study has several limitations. We analyzed only common genetic variants, so rare mutations with strong effects on lipid regulation would not appear in our dataset. Additionally, the alcohol consumption data was self-reported by participants. We tried to validate this using liver enzyme measurements, but self-reported drinking habits are never completely accurate [33]. Nevertheless, multiple lines of evidence support the clinical relevance of our findings. Similar genetic associations have been reported in independent cohorts. The biochemical markers we measured correspond to the metabolic phenotypes we observed. The proposed biological mechanisms are consistent with current understanding of lipid metabolism. Genotyping *CCDC63* variants could identify individuals at elevated risk for alcohol-related dyslipidemia. This information could inform clinical practice through more frequent lipid monitoring in genetically susceptible individuals, personalized alcohol consumption guidance based on genotype, and targeted lifestyle interventions for those at highest risk. HECTD4 (chromosome 12q24.13) lies approximately 1.25 Mb from *CCDC63* (12q24.11). In the same KARE cohort, HECTD4 associates with both alcohol consumption and lipid metabolism [34]. STRING network analysis [35] suggests these neighboring genes may jointly regulate lipid metabolism in response to alcohol. This network relationship indicates HECTD4 warrants investigation through experimental studies and mediation analysis in our dataset. Prospective validation remains necessary before clinical application. Moving forward, research should address two priorities: characterizing the molecular mechanisms of *CCDC63* function and confirming these associations across diverse populations.

## 4. Materials and Methods

### 4.1. Study Population

Korean participants were drawn from a population-based cohort initiated by the Korea National Institute of Health in 2009 [36]. After applying prespecified exclusion criteria, the final analytic cohort comprised 6655 adults aged 40–69 years. Cases were defined as individuals meeting established dyslipidemia criteria (*N* = 4327): total cholesterol ≥ 240 mg/dL, HDL cholesterol ≤ 40 mg/dL, LDL cholesterol ≥ 160 mg/dL, or triglycerides ≥ 200 mg/dL (at least one criterion met). Controls were participants without a history of dyslipidemia and not receiving lipid-lowering medications (*N* = 2328). Individuals with missing key variables, borderline lipid measurements (cutoffs predefined), or protocol noncompliance were excluded from all analyses. Healthy comparators demonstrated optimal lipid profiles characterized by total cholesterol < 200 mg/dL, high-density lipoprotein > 40 mg/dL, low-density lipoprotein < 130 mg/dL, and triglycerides < 150 mg/dL. Ethical oversight was provided by the KCDC Institutional Review Board, with documented participant consent obtained according to institutional guidelines.

### 4.2. Genetic Profiling

Genomic DNA was extracted from leukocytes isolated from peripheral venous blood. Genetic variation was assayed with the Affymetrix Genome-Wide Human SNP Array 5.0 platform (Santa Clara, CA, USA). The analysis targeted the *CCDC63* locus on chromosome 12, defined as a 10 kb interval extending 5 kb upstream and downstream of the transcriptional boundaries. Candidate single-nucleotide polymorphisms within this interval were obtained from the Korean Association REsource (KARE) dataset. Genomic coordinates were mapped to the February 2009 human genome assembly (GRCh37/hg19) based on UCSC Genome Browser annotations.

### 4.3. Data Analysis Framework

Computational analyses utilized the PLINK software, version 1.9 (https://www.cog-genomics.org/plink2, accessed on 10 February 2025), integrated with the SPSS statistical package, version 27 (IBM Corporation, Armonk, NY, USA). Association testing between *CCDC63* polymorphisms and lipid disorders employed logistic regression modeling under additive inheritance assumptions. Linear regression frameworks evaluated relationships linking significant variants with hepatic biomarkers (alanine aminotransferase, aspartate aminotransferase, and γ-glutamyl transferase) and lipid profile (high-density lipoprotein and triglycerides). Potential biases were controlled by incorporating age, sex, BMI, and residential location as covariates in all regression analyses. Participants were divided into AA homozygotes and G-variant individuals to evaluate the interaction between rs10849915 and alcohol history. Additionally, the duration of alcohol intake was partitioned into five-year categories (spanning ≤ 5 to >21 years) to observe metabolic trends over time. We quantified subgroup-specific risks for dyslipidemia through odds ratios and 95% confidence intervals. To account for multiple testing of 6 SNPs in the *CCDC63* region, Bonferroni correction was applied with an adjusted significance threshold of *p* < 0.0083 (0.05/6). Sensitivity analyses were performed with additional adjustment for smoking status to assess the robustness of genetic associations (Supplementary Table S2).

## 5. Conclusions

Our findings provide evidence that *CCDC63* is a critical genetic factor modulating metabolic homeostasis in a manner strictly contingent upon alcohol consumption. We observed that the genetic influence on dyslipidemia and its associated biomarkers is restricted to the drinking population, indicating a profound gene–environment interaction. This study clarifies the paradoxical metabolic signature of *CCDC63*—reduced γ-GTP levels coupled with an increased risk of dyslipidemia—which reveals the multifaceted impact of alcohol on systemic lipid homeostasis. These insights not only identify *CCDC63* as a potential biomarker for cardiovascular assessment in alcohol-consuming groups but also advocate for a lifestyle-integrated approach in human genomic studies.

## Figures and Tables

**Table 1:** Characteristics of the study participants with the Korean association REsource (KARE) cohort.

Characteristics *	Quantitative Trait Analysis	Case Control Analysis **		
		Normal	Dyslipidemia	*p*-Value ***
Number of Subjects	6655	2328	4327	
Sex [Male(%)/Female(%)]	3177(47.7)/3478(52.3)	908(39.0)/1420(61.0)	2269(52.4)/2058(47.6)	<0.001
Age (M Years ± SD)	52.10 ± 8.92	51.12 ± 9.08	52.68 ± 8.79	0.013
Body Mass Index (BMI) (M kg/m^2^ ± SD)	24.56 ± 3.14	23.31 ± 3.12	25.22 ± 2.94	0.003
Total Cholesterol (Tchl) (M mg/dL ± SD)	186.43 ± 36.96	168.71 ± 19.12	195.97 ± 40.56	<0.001
Triglyceride (TG) (M mg/dL ± SD)	154.99 ± 74.94	99.41 ± 24.44	184.90 ± 75.90	<0.001
High-Density Lipoprotein (HDL) Cholesterol (M mg/dL ± SD)	43.32 ± 9.78	50.71 ± 7.94	39.35 ± 8.24	0.001
Low Density Lipoprotein (LDL) Cholesterol (M mg/dL ± SD)	112.11 ± 33.36	98.12 ± 18.92	119.64 ± 36.84	<0.001

* Abbreviations: M, mean; SD, standard deviation. Data are shown as mean ± SD for continuous variables or n (%) for categorical variables. ** Normal group: Tchl < 200 mg/dL, HDL > 40 mg/dL, LDL < 130 mg/dL, TG < 150 mg/dL (all four criteria met); dyslipidemia group: Tchl ≥ 240 mg/dL or HDL ≤ 40 mg/dL or LDL ≥ 160 mg/dL or TG ≥ 200 mg/dL (≥1 criterion met) *** Statistical comparisons between groups were performed using two-tailed Student’s *t*-test (*p* < 0.05).

**Table 2:** *CCDC63* genetic polymorphisms associated with dyslipidemia susceptibility.

No.	SNP	A1	A2	Function	MAF		Dyslipidemia	
							(Controls 2328: Cases 4327)	
					Controls	Cases	OR (95% CI)	Add *p*
1	rs2283348	T	C	Intron	0.3877	0.3768	0.95 (0.89~1.03)	0.2162
2	rs10219717	T	C	Intron	0.3899	0.3798	0.96 (0.89~1.03)	0.2520
3	rs756838	T	A	Intron	0.3901	0.3799	0.96 (0.89~1.03)	0.2457
4	rs2238149	G	A	Intron	0.1554	0.1749	1.15 (1.05~1.27)	**0.0043**
5	rs10849915	G	A	Intron	0.1645	0.1852	1.15 (1.05~1.27)	**0.0032**
6	rs11065756	A	G	Intron	0.1634	0.1844	1.16 (1.05~1.27)	**0.0026**

Logistic regression analysis under an additive genetic model adjusting for age, sex, body mass index, and region. Abbreviations: SNP, single nucleotide polymorphism; A1, minor allele; A2, major allele; MAF, minor allele frequency; OR, odds ratio; CI, confidence interval; Add *p*, *p*-value under additive genetic model. Bold values highlight *p* < 0.05.

**Table 3:** Comparison of rs10849915 associations with alcohol consumption in previous genome-wide association study.

Phenotype	Previous Studies				Replication in Koreans		
	EA	β ± SE, OR (95% CI)	*p*-Value ***	Research Study	EA	β ± SE, OR (95% CI)	*p*-Value ***
Alcohol Consumption	G	−0.55 ± 0.05	1.0 × 10^−23^	Inkyung Baik et al.	G	−0.31 ± 0.02	1.31 × 10^−60^
	C	0.04	4.0 × 10^−28^	Eric Jorgenson et al.	NA		

Comparison of rs10849915 effect sizes across population. All analyses were adjusted for age, sex, and body mass index. Previous studies examined alcohol consumption as continuous variable (drinks per week or grams per day). Current study validated associations in Korean cohort using identical phenotype definition. Abbreviations: β, regression coefficient; CI, confidence interval; EA, effect allele; NA, not available; OR, odds ratio; SE, standard error. *** Asterisk mark indicates genome-wide significance (*p* < 5 × 10^−8^ ).

**Table 4:** Three *CCDC63* polymorphisms associated with alcohol-related hepatic enzymes and serum lipid profiles.

Traits	rs10849915		rs11065756		rs2238149	
	Effect Size	*p*-Value	Effect Size	*p*-Value	Effect Size	*p*-Value
	(β ± s.e)		(β ± s.e)		(β ± s.e)	
Alcohol-Related Liver Enzyme Levels						
ALT (U/L)	−1.18 ± 0.51	**0.02214**	−1.13 ± 0.52	**0.02966**	−0.95 ± 0.53	0.07174
AST (U/L)	−1.20 ± 0.37	**0.00125**	−1.19 ± 0.37	**0.00134**	−1.16 ± 0.38	**0.00220**
γ-GTP (U/L)	−6.99 ± 1.29	**5.68 × 10^−8^**	−7.24 ± 1.29	**2.07 × 10^−8^**	−7.55 ± 1.31	**8.67 × 10^−8^**
Serum lipid levels						
HDL (mg/dL)	−0.87 ± 0.29	**0.00244**	−0.91 ± 0.29	**0.00148**	−0.79 ± 0.29	**0.00704**
TG (mg/dL)	−3.49 ± 2.19	0.11080	−3.68 ± 2.20	0.09409	−3.93 ± 2.24	0.07923

Linear regression analysis under additive genetic model adjusting for age, sex, body mass index, and residential area. Effect sizes (β) represent change in trait levels per additional minor allele. Hepatic enzymes measured in U/L; lipid parameters measured in mg/dL. Abbreviations: ALT, alanine aminotransferase; AST, aspartate aminotransferase; γ-GTP, gamma-glutamyl transpeptidase; HDL, high-density lipoprotein cholesterol; TG, triglycerides. Bold values indicate *p* < 0.05.

**Table 5:** rs10849915 genotype-stratified analysis of alcohol exposure duration and dyslipidemia risk.

	AA Genotype (*N* = 2554)		AG/GG Genotype (*N* = 821)	
	OR (95% CI)	*p*-Value ***	OR (95% CI)	*p*-Value ***
Sex	0.41 (0.34~0.50)	<0.001	1.17 (0.59~2.33)	0.646
Age	1.01 (1.00~1.02)	0.264	1.13 (1.08~1.18)	<0.001
BMI	1.33 (1.29~1.37)	<0.001	1.29 (1.15~1.44)	<0.001
γ-GTP	1.00 (1.00~1.01)	<0.001	1.00 (1.00~1.00)	<0.001
Alcohol Consumption				
<5	1 (ref)	<0.001	1 (ref)	0.05
6~10	1.10 (0.76~1.58)	0.062	1.29 (0.70~2.40)	0.04
11~15	1.19 (0.81~1.77)	0.038	1.44 (0.75~2.79)	0.03
16~20	2.03 (1.44~2.85)	<0.001	1.55 (0.84~2.84)	0.02
>21	1.92 (1.47~2.49)	<0.001	1.43 (0.93~2.20)	0.01

Logistic regression analysis stratified by rs10849915 genotype. Alcohol consumption duration categories: ≤5 years (reference), 6–10 years, 11–15 years, 16–20 years, and >21 years. Models adjusted for age, sex, body mass index, and γ-glutamyl transferase levels. Odds ratios (OR) with 95% confidence intervals (CI) represent dyslipidemia risk relative to ≤5 years exposure within each genotype group. *p*-values calculated from Wald test. Abbreviations: BMI, body mass index; CI, confidence interval; γ-GTP, gamma-glutamyl transferase; OR, odds ratio. AG and GG carriers were combined due to the low frequency of GG homozygotes and similar dyslipidemia prevalence between these groups. *** Asterisks denote statistical significance (*p* < 0.05).

**Table 6:** Sex-Specific effects of rs10849915 on dyslipidemia and metabolic traits.

Traits	Male		Female	
	Effect Size	*p*-Value	Effect Size	*p*-Value
	β ± SE, OR (95% CI)		β ± SE, OR (95% CI)	
Dyslipidemia	1.24 (1.07~1.43)	**0.00340**	1.07 (0.95~1.22)	0.27370
Alcohol Consumption	−0.40 ± 0.02	**2.71 × 10^−74^**	−0.23 ± 0.02	**1.66 × 10^−22^**
Clinical Indicators of Alcohol Consumption				
ALT (U/L)	−1.45 ± 0.94	**0.01223**	−0.97 ± 0.45	**0.03138**
AST (U/L)	−1.98 ± 0.64	**0.00215**	−0.51 ± 0.38	0.18140
γ-GTP (U/L)	−13.02 ± 2.51	**2.16 × 10^−7^**	−1.60 ± 0.65	**0.01461**
Dyslipidemia Clinical Indicators				
HDL (mg/dL)	−1.86 ± 0.33	**0.00000**	0.06 ± 0.45	0.90240
TG (mg/dL)	−9.83 ± 3.57	**0.00591**	2.19 ± 2.55	0.39110

Logistic regression (for dyslipidemia) and linear regression (for continuous traits) under additive genetic model. Analyses performed separately in males (*N* = 3177) and females (*N* = 3478). Models adjusted for age, body mass index, and residential area. Effect sizes shown as odds ratios (OR) for dyslipidemia and regression coefficients (β ± SE) for continuous variables. Abbreviations: ALT, alanine aminotransferase; AST, aspartate aminotransferase; γ-GTP, gamma-glutamyl transferase; HDL, high-density lipoprotein cholesterol; TG, triglycerides. Bold values indicate *p* < 0.05.

## Data Availability

The original contributions presented in this study are included in the article/Supplementary Material. Further inquiries can be directed to the corresponding author.

## Supplementary Materials

The following supporting information can be downloaded at: https://www.mdpi.com/article/10.3390/ijms27052134/s1.
