# Supplementary material for: Genetic Influence of CCDC63 Polymorphisms on Alcohol-Induced Dyslipidemia in a Korean Cohort
Source: Int J Mol Sci. 2026 Feb 25;27(5):2134. doi: 10.3390/ijms27052134 (PMC12984777; doi:10.3390/ijms27052134)
Supplement: Supplementary file 1 [file ijms-27-02134-s001.zip › Table S1.pdf]

Supplementary Materials

Table S1. Association between *CCDC63* variants and metabolic traits stratified by alcohol consumption

SNP	Phenotype	Non-drinkers (n=3,109)		Ever-Drinkers (n=3,485)	
		**OR/β (95% CI)**	***P-value***	**OR/β (95% CI)**	***P-value***
rs10849915	Dyslipidemia (OR)	1.12 (0.99~1.28)	0.071	1.23 (1.06~1.42)	**0.007**
	γ-GTP (β)	0.94 (-0.46~2.35)	0.187	-8.08 (-13.0~-3.16)	**0.001**
	HDL (β)	-0.07 (-0.63~0.48)	0.793	-1.42 (-2.11~-0.73)	**<0.001**
rs11065756	Dyslipidemia (OR)	1.13 (1.00~1.29)	0.057	1.23 (1.06~1.43)	**0.008**
	γ-GTP (β)	0.97 (-0.43~2.37)	0.176	-8.72 (-13.7~-3.76)	**0.001**
rs2238149	Dyslipidemia (OR)	1.09 (0.96~1.24)	0.185	1.26 (1.08~1.47)	**0.003**
	γ-GTP (β)	0.76 (-0.66~2.19)	0.293	-8.46 (-13.5~-3.44)	**0.001**

P-values were calculated using logistic regression for dyslipidemia and linear regression for metabolic markers, adjusted for age and sex. The contrast between non-drinkers and ever-drinkers highlights the gene-environment interaction between *CCDC63* and alcohol exposure. Bold values indicate statistical significance ($P < 0.05$). Ever drinkers include current drinkers (n=3,038) and former drinkers (n=447). Non-drinkers represent individuals who reported never consuming alcohol. Abbreviations: OR, odds ratio; CI, confidence interval; γ-GTP, gamma-glutamyl transpeptidase; HDL, high-density lipoprotein cholesterol.
