# Supplementary material for: Genetic Influence of CCDC63 Polymorphisms on Alcohol-Induced Dyslipidemia in a Korean Cohort
Source: Int J Mol Sci. 2026 Feb 25;27(5):2134. doi: 10.3390/ijms27052134 (PMC12984777; doi:10.3390/ijms27052134)
Supplement: Supplementary file 1 [file ijms-27-02134-s001.zip › Table S2.pdf]

Supplementary Materials

Table S2. Association of *CCDC63* variants with dyslipidemia before and after adjustment for smoking

SNP	A1	A2	Function	Model 1 (without smoking)		Model 2 (with smoking)	
				OR (95% CI)	*P*	OR (95% CI)	*P*
rs2238149	G	A	intron	1.15 (1.05–1.27)	0.0043	1.19 (1.07–1.32)	**0.0013**
rs10849915	G	A	intron	1.15 (1.05–1.27)	0.0032	1.19 (1.08–1.32)	**0.0007**
rs11065756	A	G	intron	1.16 (1.05–1.27)	0.0026	1.20 (1.08–1.33)	**0.0006**

Logistic regression analysis under an additive genetic model. Model 1 adjusted for age, sex, body mass index, and residential area. Model 2 additionally adjusted for smoking status (never, former, current). Odds ratios (ORs) and 95% confidence intervals (CIs) were estimated using logistic regression analyses. Following adjustment for smoking status, the associations between *CCDC63* variants and dyslipidemia remained statistically significant, with effect sizes slightly increased. Abbreviations: SNP, single nucleotide polymorphism; A1, minor allele; A2, major allele; OR, odds ratio; CI, confidence interval. Bold values indicate $P < 0.05$.
