# Supplementary material for: Genetic Influence of CCDC63 Polymorphisms on Alcohol-Induced Dyslipidemia in a Korean Cohort
Source: Int J Mol Sci. 2026 Feb 25;27(5):2134. doi: 10.3390/ijms27052134 (PMC12984777; doi:10.3390/ijms27052134)
Supplement: Supplementary file 1 [file ijms-27-02134-s001.zip › Table S3.pdf]

Supplementary Materials

Table S3. Alcohol consumption and smoking characteristics of the study participants

Characteristics	Total	Case-control analysis		*P*-value
		Normal	Dyslipidemia	
**Alcohol consumption**				
Current drinker (%)				
Never drinker	3,109 (47.1)	1,091 (35.1)	2,018 (64.9)	**<0.001**
Former drinker	447 (6.8)	98 (21.9)	349 (78.1)	**<0.001**
Current drinker	3,038 (46.1)	1,117 (36.8)	1,921 (63.2)	**<0.001**
Drinking frequency (week, %)				
< 1	904 (35.1)	325 (36.0)	579 (64.0)	0.978
1 - 3	1,116 (43.3)	372 (33.3)	744 (66.7)	**0.044**
≥ 4	556 (21.6)	223 (40.1)	333 (59.9)	**0.017**
Total alcohol intake (g/day ± SD)	20.1 ± 27.8	18.7 ± 26.3	20.9 ± 28.6	**0.039**
Soju drinker (%)	2,611 (85.9)	934 (35.8)	1,677 (64.2)	**0.006**
Beer drinker (%)	1,274 (41.9)	488 (38.3)	786 (61.7)	0.114
Makgeolli drinker (%)	321 (10.6)	130 (40.5)	191 (59.5)	0.134
Wine drinker (%)	159 (5.2)	60 (37.7)	99 (62.3)	0.778
Spirits drinker (%)	283 (9.3)	65 (23.0)	218 (77.0)	**<0.001**

**Drinking duration (years, %)**				
≤ 5	375 (11.0)	169 (45.1)	206 (54.9)	**<0.001**
6 – 10	289 (8.5)	121 (41.9)	168 (58.1)	**0.006**
11 – 15	229 (6.7)	91 (39.7)	138 (60.3)	**0.005**
16 – 20	401 (11.8)	125 (31.2)	276 (68.8)	0.179
≥ 21	2,101 (61.9)	675 (32.1)	1,426 (67.9)	**<0.001**
**Smoking status (%)**				
Never	3,834 (58.4)	1,515 (39.5)	2,319 (60.5)	**<0.001**
Former	1,023 (15.6)	280 (27.4)	743 (72.6)	**0.006**
Current	1,709 (26.0)	495 (29.0)	1,214 (71.0)	**<0.001**

Data are presented as mean ± standard deviation (SD) for continuous variables and as number (percentage) for categorical variables. Percentages for alcohol type (soju, beer, makgeolli, wine, and spirits) were calculated among current drinkers only. Total alcohol intake (g/day) was calculated as the sum of ethanol intake from all alcoholic beverages based on standardized ethanol content. Drinking frequency represents the number of drinking occasions per week, and drinking duration indicates the total number of years of alcohol consumption. Smoking status was categorized as never, former, or current smoker. *P*-values were calculated using Student's *t*-test for continuous variables and the chi-square test for categorical variables. Bold values indicate $P < 0.05$.
