# Supplementary material for: Genetic Influence of CCDC63 Polymorphisms on Alcohol-Induced Dyslipidemia in a Korean Cohort
Source: Int J Mol Sci. 2026 Feb 25;27(5):2134. doi: 10.3390/ijms27052134 (PMC12984777; doi:10.3390/ijms27052134)
Supplement: Supplementary file 1 [file ijms-27-02134-s001.zip › Table S4.pdf]

Supplementary Materials

Table S4. Genotype distribution and prevalence of dyslipidemia according to rs10849915

Genotype	N (%)	Dyslipidemia (%)
AA	4,484 (67.7)	2,861 (63.8)
GA	1,914 (28.9)	1,290 (67.4)
GG	221 (3.3)	152 (68.8)

Data are presented as number (percentage). Percentages of dyslipidemia represent the proportion of individuals with dyslipidemia within each genotype group. Dyslipidemia prevalence showed a dose-dependent increase with the number of G alleles: 63.8% (AA) → 67.4% (AG) → 68.8% (GG).
